# Has the NTD Community Neglected Evidence-Based Policy?

**DOI:** 10.1371/journal.pntd.0002238

**Published:** 2013-07-11

**Authors:** Sukrti Nagpal, David Sinclair, Paul Garner

**Affiliations:** 1 Royal Surrey County Hospital, Guildford, Surrey, United Kingdom; 2 Liverpool School of Tropical Medicine, Liverpool, United Kingdom; Lindsley F. Kimball Research Institute, New York Blood Center, United States of America

## Evidence-Informed Policy?

Over the past decade, systematic reviews and explicit evidence-based approaches have replaced expert opinion as the basis for health policy [1]. In developing countries, the high disease burden coupled with limited financial resources for health requires governments, donors, and the public to choose between competing public health and clinical care options, and increasingly they turn to “evidence” to inform these decisions.

Systematic reviews of the benefits and harms of different policies and treatments are one of the core sources of evidence, providing concise summaries of the available research about effects [2]. Well-conducted systematic reviews aim to minimize bias in presenting and interpreting results. This can arise due to intentional or unintentional selective studies inclusion, selective reporting, uncritical reading of poorly conducted trials, and incorrect inferences from the data across studies [3]. Alongside evidence on effects, decision making requires other information including cost, disease burden, and the acceptability and feasibility of the policy options. Nevertheless, data on effects is fundamental, because if something does not work, it will not impact on health, and it is not cost-effective, irrespective of whether the drug is cheap or expensive.

As members of the Cochrane Infectious Diseases Group, we have, over the past twenty years, seen steady growth in the demand for systematic reviews to inform international and national policy decisions in infectious diseases. Our reviews have been used by policy makers as they have made recommendations to scale-up impregnated mosquito nets [4], reintroduce amodiaquine for malaria [5], change the formula of ORS [6], and switch to artesunate for severe malaria [7]. However, not all our systematic reviews support current policies; some identify research gaps, and some cast doubt on the benefits of the interventions. These gaps may indicate further research is required before these interventions can be recommended.

The most complicated policy situations are those in which there is limited evidence of public health benefit, yet statements made by experts recommend the policy. If indeed the expert opinion is wrong, then the continued delivery of the intervention may waste public resources, or fail to bring about all of the promised benefits. One such example of a current debate is in routine deworming of all schoolchildren in areas where intestinal helminths occur. The Cochrane review, in light of current guidelines, advocacy, and policies, represents an area where assumed benefit by expert panels is by no means supported by quite a lot of available, reliable research [8]. Whatever the outcome of future recommendations from the World Health Organization and others is, what is important is that independent syntheses by groups external to the advocacy provide reliable summaries that can be considered in decision making.

We are concerned that the neglected tropical disease (NTD) academic community has been slow to engage in evidence-informed policy and debate, and may be falling behind international best practice. This is borne out of a concern that NTDs are important diseases that need treatments, but that international policies need to stay in line with current international expectations of evidence-informed policy to avoid being discredited. To look for evidence to confirm or refute these concerns, we used appropriate systematic methods (Text S1), and present our interpretation of these data as a viewpoint at the request of *PLOS NTDs* editors.

## Influential Papers in NTD Policy and the Evidence They Cite

To evaluate the use of evidence in formulating current policies, we first created a database of NTD citations by searching MEDLINE for all articles containing “neglected,” “neglected disease,” or “neglected tropical disease” in the title or abstract, up to June 2012. From this database, we used the Science Citation Index to identify the ten most commonly cited articles, conducted a brief content analysis of these, and examined how they referenced systematic reviews of effects and randomized controlled trials to support the policies they advocated.

The complete findings of this analysis are available in the supplementary material published with this Viewpoint (see Text S1). Of the ten articles, three focus on disease burden and do not strongly advocate for any particular intervention [9]–[11]. The remaining seven promote mass drug administration, with four drugs, for between five and seven diseases [12]–[18]. They cite between them a total of two systematic reviews and 12 randomized trials. Our interpretation of the analysis is that:

- The top-cited advocacy articles have assumed the effectiveness of mass drug administration for all diseases encompassed;
- Citation of randomized controlled trials is highly selective, and usually does not relate to the main thrust of the campaigns;
- Citation of Cochrane reviews is nonexistent, although at least 12 relevant reviews were available when the advocacy articles were published.

## What Evidence Is Available?

There are now 67 Cochrane reviews on NTDs available in the Cochrane library. While the effectiveness of some interventions and programmes has been demonstrated, the effectiveness of others is unclear. For example:

- For soil-transmitted helminth infection, the drugs clear the infection and improve health in infected individuals; but routine mass deworming of children probably doesn't have strong population benefits (42 included studies, 65,168 participants) [8], and routine deworming of pregnant women has not been shown to improve maternal or birth outcomes (three included studies, 1,329 participants) [19], [20].
- For schistosomiasis, praziquantel appears to be effective at clearing infection with *S. mansoni* (52 included studies, 10,269 participants) [21] and *S. haematobium* (24 included studies, 6,315participants) [22], but the relative benefits and harms of mass population treatments need to be reviewed.
- For filariasis, diethylcarbamazine has been shown to reduce microfilariae in individuals, and in communities through medicated-salt programmes (21 included studies, >100,000 participants) [23]; but the current evidence for albendazole does not demonstrate an effect that is any different from placebo (seven included studies, 6,997 participants) [24].
- For onchocerciasis, ivermectin has been shown to be effective at preventing some forms of eye damage in individuals and mass-treated populations, but a reduction in eventual blindness has not been adequately proven (four included studies, 5,399 participants) [25].
- For trachoma, antibiotics have been shown to be effective at reducing prevalence in community programmes (14 included studies, 3,587 participants) [26], but face washing programmes have not (two included studies, 2,560 participants) [27].

## Our View

The “neglected tropical disease” movement, like many areas of tropical medicine, has a few powerful advocates driving the international agenda. This advocacy-based approach has been highly successful at raising the profile of these important tropical diseases and instrumental in the development of policies and donor funds for implementing disease control programmes.

However, we would now encourage the academic NTD field to be reflective and critical about the current policies, practice, and impact. To date, there appears to have been little use of systematic reviews and only selective use of individual trials when formulating these policies. The most cited policy articles appear to assume the effectiveness of mass drug administration, rather than present a policy option supported by a reliable evidence base. While some of these policies concerned drugs already clearly established as effective in individuals (such as praziquantel for schistosomiasis) and in communities (such as DEC-treated salt for preventing filariasis), others remain less clear, particularly when used in mass administration programmes.

Our concern is that if we ignore this mismatch, it could threaten the long-term credibility of the programmes the advocates are promoting. We would argue that to be credible long term, to reassure donors that money is being spent effectively, and to ensure the best possible outcomes for people living in endemic areas, the field should be explicit and transparent about the link between the policies being advocated and a reliable evidence base.

NTD advocacy started with pleas for new drugs, and researchers need to continue to develop, evaluate, and test new interventions and treatment combinations through properly conducted randomized controlled trials [28]. For interpretation and integration of this research into reliable health policies, this necessarily requires their incorporation into independent, critical, and reliable systematic reviews. We hope that through dialogue and debate, a new agenda of research priorities will emerge, both for randomized controlled trials evaluating drug efficacy in individual diseases and for pragmatic implementation trials and health services research to examine the impacts of these programmes on community health. Reviews conducted by independent specialists in collaboration with topic specialists should be part of helping achieve global consensus as the science in the field moves forward.

## Supporting Information

Text S1 **A systematic appraisal of use of evidence in the most highly cited NTD literature: methods and results.** The authors created a database of trials that wrote about “neglected tropical diseases” through careful bibliometric analysis; they then took the ten most cited articles and carried out a content analysis. This examined what the main message of the paper was; who the authors were; and how they cited evidence, particularly randomized controlled trials and systematic reviews. This was then compared with systematic reviews that were available at the time of publication, and inferences drawn.
(DOCX)
