# Supplementary material for: Has the NTD Community Neglected Evidence-Based Policy?
Source: PLoS Negl Trop Dis. 2013 Jul 11;7(7):e2238. doi: 10.1371/journal.pntd.0002238 (PMC3708801; doi:10.1371/journal.pntd.0002238)
Supplement: Text S1 — A systematic appraisal of use of evidence in the most highly cited NTD literature: methods and results. The authors created a database of trials that wrote about “neglected tropical diseases” through careful bibliometric analysis; they then took the ten most cited articles and carried out a content analysis. This examined what the main message of the paper was; who the authors were; and how they cited evidence, particularly randomized controlled trials and systematic reviews. This was then compared with systematic reviews that were available at the time of publication, and inferences drawn. (DOCX) [file pntd.0002238.s001.docx]

### Supplementary material:

### A systematic appraisal of use of evidence in the most highly cited NTD literature: methods and results

Sukrti Nagpal^1^*, David Sinclair^2^, Paul Garner^2^

**^1^** Royal Surrey County Hospital, Guildford, Surrey, United Kingdom

**^2^** Liverpool School of Tropical Medicine, Liverpool, United Kingdom

* E-mail: [sukrti.nagpal@gmail.com](mailto:sukrti.nagpal@gmail.com)

18 April 2013

Supplementary document published on PLOS Neglected Tropical Diseases accompanying the article: Nagpal S, Sinclair D, Garner P. Has the NTD Community Neglected Evidence-Based Policy?

### Objective

To examine the key academic policy articles in Neglected Tropical Diseases, and how they use contemporary forms of evidence-informed literature.

### Methods

We created an NTD database by searching MEDLINE for all articles containing ‘neglected’, ‘neglected disease’ or ‘neglected tropical disease’ in the title or abstract, up to June 2012. We recorded the number of unique articles published each year using these terms, and tracked the working definition of which diseases were included and excluded.

From this database we used the Science Citation Index to identify the ten most commonly cited articles, and conducted a brief content analysis on the aims and objectives of the articles, and documented references to systematic reviews of effects and randomized controlled trials.

To identify available systematic reviews in this area, we used the PLOS Medicine definition [[1](#_ENREF_1)] of diseases that constitute NTDs and conducted searches of MEDLINE and the Cochrane library. The findings of the systematic reviews were then compared to the policies advocated by the top ten articles.

For each of the ten articles we then extracted information relating to the following criteria:

1. The title and number of citations in the Science Citation Index.
2. The type of article; be it review, narrative, or viewpoint.
3. The policies promoted within the article, and the number of drugs and disease advocated.
4. Types of evidence referred to, and
5. Whether the article considered harms.

### Results

The ten articles were published between 2005 and 2009, and all authored by an overlapping group of opinion leaders in the field (see table 1). Six articles are clearly identified as ‘review’ articles and take the form of traditional ‘narrative’ reviews[[2](#_ENREF_2),[3](#_ENREF_3),[4](#_ENREF_4),[5](#_ENREF_5),[6](#_ENREF_6),[7](#_ENREF_7)], two are labeled as expert opinion or ‘viewpoints’[[8](#_ENREF_8),[9](#_ENREF_9)], and for two there was no clear identifier but they could fall into either group[[10](#_ENREF_10),[11](#_ENREF_11)]. None contained a methods section which explicitly stated the questions being addressed, or the methods used to find and appraise relevant literature, and so none could be considered systematic reviews.

All ten articles discussed the disease burden and overlapping epidemiology of the NTDs. Seven advocated for future NTD control through mass drug administration, through ‘integrated vertical programmes’ targeting 5-7 diseases (schistosomiasis, lymphatic filariasis, ascariasis, trichuriasis, and hookworm +/- onchocerciasis and trachoma), using 3-4 drugs (praziquantel, ivermectin or DEC, and albendazole or mebendazole, +/- azithromycin.

The table shows our analysis, and these are our observations:

1. None of the articles reference Cochrane systematic reviews despite the availability of 12 reviews relevant to the control of these seven diseases at the time these advocacy articles were published.
2. Only one systematic review of drug efficacy is cited (Tisch 2005) [[12](#_ENREF_12)]: This review synthesizes 57 randomized controlled trials evaluating the effects of DEC, ivermectin and albendazole on microfilaria prevalence. The review concludes that combinations containing DEC are most effective.
3. One systematic review of mass drug administration is cited (Reddy 2007)[[13](#_ENREF_13)]: This review synthesizes the results of 27 randomized trials involving MDA of at least two drugs. The review concentrates on effects on parasite prevalence and does not report clinical or nutritional outcomes.
4. Aside from Hotez 2006 these papers cite less than two RCTs each as evidence of effect.
5. Hotez 2006 selectively references 7 randomized trials reporting positive findings, while the negative trials that exist are ignored.
6. None of the six review articles present efficacy estimates for the individual drugs, and none adequately consider any potential adverse effects.
7. Similarly, none present community level effect estimates for MDA programmes, and none adequately consider the potential harms of mass drug administration.

### Conclusions

The six most cited policy review articles present an argument for mass drug administration based on the ‘size of the problem’ (the estimated disease burden), and ‘convenience’ with drug companies donating drugs, and the possibility to piggyback on existent programmes. None of the articles presented policy options supported by evidence presented explicitly and transparently.

**Table S1 Content analysis of the top ten most commonly cited NTD articles**

| **Article ID** | **No. of cites** | **Article type** | **Policy promoted** | **How many drugs?** | **For how many diseases?** | **Evidence cited for benefits of drug treatment for:** | | | | | | **Considers potential harms** | |
| --- | --- | --- | --- | --- | --- | --- | --- | --- | --- | --- | --- | --- | --- |
|  |  |  |  |  |  | **Infected individuals** | | | **Unscreened populations** | | | **Individual drugs** | **Mass Drug Administration** |
|  |  |  |  |  |  | CR | SR | RCT | CR | SR | RCT |  |  |
| **Molyneux 2005** | 150 | Not stated | MDA^1^ | 4 | 7 | 0 | 0 | 0^2^ | 0 | 0 | 0 | No | No^3^ |
| **Hotez 2006** | 199 | Not stated | MDA^1^ | 4 | 7 | 0 | 0 | 3^4,5,6^ | 0 | 0 | 4^7,8,9,10^ | No | No^11^ |
| **Lammie 2006** | 87 | Review | MDA^1^ | 3 | 5 | 0 | 1^12^ | 0 | 0 | 0^13^ | 2^14,15^ | No^16^ | No^3^ |
| **Hotez 2007** | 330 | Review | MDA^1^ | 4 | 7 | 0 | 0 | 0 | 0 | 0 | 0 | No | No |
| **Hotez 2008a** | 97 | Review | MDA^1^ | 4 | 7 | 0 | 0 | 0 | 0 | 0 | 1^17^ | No | No |
| **Hotez 2008b** | 182 | Review | MDA^1^ | 4 | 7 | 0 | 0 | 1^18,2^ | 0 | 0 | 1^17^ | No | No^11^ |
| **Hotez 2009a** | 107 | Viewpoint | MDA^1^ | 4 | 7 | 0 | 0 | 1^5^ | 0 | 1^19^ | 0 | No | No |
| **Engels 2006** | 54 | Opinion | These articles are concerned with disease burden and do not strongly advocate for a particular intervention | | | | | | | | | | |
| **Mathers 2007** | 86 | Review |  |  |  |  |  |  |  |  |  |  |  |
| **Hotez 2009b** | 91 | Review |  |  |  |  |  |  |  |  |  |  |  |

^1^ All articles advocate integration of disease specific vertical mass drug administration (MDA) programmes to target several diseases with a combination of drugs.

^2^ One additional RCT was referenced but this was evaluating doxycycline for treating filariasis (Taylor 2005).[[14](#_ENREF_14)]

^3^ Briefly considers the potential effect on drug resistance of MDA.

^4^ A RCT where Kenyan school boys received either albendazole or placebo. Outcomes included physical fitness, parasite rates, growth rates and Hb concentration. (Stephenson 1989-1993)[[15](#_ENREF_15),[16](#_ENREF_16),[17](#_ENREF_17),[18](#_ENREF_18)]

^5^ A RCT comparing albendazole and placebo for treating moderate to high loads of Trichuris infection and reporting effects on cognitive function. (Nokes 1992)[[19](#_ENREF_19),[20](#_ENREF_20)]

^6^ A RCT comparing Praziquantel and placebo for treating S. japonicum and reporting effects on cognitive function. (Nokes 1999)[[21](#_ENREF_21)]

^7^ A RCT comparing MDA with albendazole plus Praziquantel versus placebo for school aged children and reporting effects on prevalence and haemoglobin. (Beasley 1999)[[22](#_ENREF_22)]

^8^ A RCT comparing MDA with albendazole or Praziquantel or both versus vitamin B for school aged children and reporting effects on haemoglobin and ferritin. (Bhargava 2003)[[23](#_ENREF_23)]

^9^ A RCT comparing MDA with mebendazole versus placebo for preschool children and reporting effects on appetite, anaemia& growth. (Stoltzfus 2004)[[24](#_ENREF_24)]

^10^ A RCT comparing MDA with albendazole versus placebo for pregnant mothers and reporting effects on immunological outcomes to BCG vaccinations in infants and mothers. (Elliott 2005)[[25](#_ENREF_25)]

^11^ Briefly mentions potential harms of MDA, however does not discuss in detail.

^12^ A systematic review of trials evaluating drugs used to treat filariasis and reporting effects on microfilaria prevalence (Tisch 2005).[[12](#_ENREF_12)]

^13^ One additional reference is labeled as a ‘review article’ but would not meet the international definition of a systematic review as it does not contain a methods section. (Gyapong 2005)[[26](#_ENREF_26)]

^14^ A RCT comparing diethylcarbamazine (DEC) + ivermectin vs. DEC alone to children in PNG reporting outcomes of reservoir of microfilariae, severity of lymphatic filariasis abnormalities, incidence of new infection and adverse events. (Bockarie 2002)[[27](#_ENREF_27)]

^15^ A RCT comparing MDA with mebendazole +/- levimasole versus placebo for school children and reporting effects on prevalence and intensity of infection of soil transmitted helminths. (Albonico 2003)[[28](#_ENREF_28)]

^16^ Briefly considers the safety of co-administration of drugs.

^17^ A RCT comparing MDA with mebendazole versus placebo for pregnant women and reporting effects on birth weight and maternal anaemia (Larocque 2006) [[29](#_ENREF_29)]

^18^ A RCT comparing albendazole with mebendazole for treating hookworm (Flohr 2007) [[30](#_ENREF_30)]

^19^ A systematic review of RCTs comparing drug administration targeting at least two NTDs with placebo and reporting effects on prevalence (Reddy 2007)[[13](#_ENREF_13)]

### References:

1. Journal Scope. PLoS Negl Trop Dis.

2. Lammie PJ, Fenwick A, Utzinger J (2006) A blueprint for success: integration of neglected tropical disease control programmes. Trends in Parasitology 22: 313-321.

3. Hotez PJ, Molyneux DH, Fenwick A, Kumaresan J, Sachs SE, et al. (2007) Control of Neglected Tropical Diseases. New England Journal of Medicine 357: 1018-1027.

4. Hotez PJ, Bottazzi ME, Franco-Paredes C, Ault SK, Periago MR (2008) The Neglected Tropical Diseases of Latin America and the Caribbean: A Review of Disease Burden and Distribution and a Roadmap for Control and Elimination. PLoS Negl Trop Dis 2: e300.

5. Hotez PJ (2008) Helminth infections: the great neglected tropical diseases. The Journal of Clinical Investigation 118: 1311-1321.

6. Mathers C (2007) Measuring the Burden of Neglected Tropical Diseases: The Global Burden of Disease Framework. PLoS Neglected Tropical Diseases 1.

7. Hotez PJ, Kamath A (2009) Neglected Tropical Diseases in Sub-Saharan Africa: Review of Their Prevalence, Distribution, and Disease Burden. PLoS Negl Trop Dis 3: e412.

8. Peter JH, Alan F, Lorenzo S, David HM (2009) Rescuing the bottom billion through control of neglected tropical diseases. The Lancet 373: 1570-1575.

9. Engels D, Savioli L (2006) Reconsidering the underestimated burden caused by neglected tropical diseases. Trends in Parasitology 22: 363-366.

10. Molyneux DH, Hotez PJ, Fenwick A (2005) “Rapid-Impact Interventions”: How a Policy of Integrated Control for Africa's Neglected Tropical Diseases Could Benefit the Poor. PLoS Med 2: e336.

11. Hotez PJ, Molyneux DH, Fenwick A, Ottesen E, Ehrlich Sachs S, et al. (2006) Incorporating a Rapid-Impact Package for Neglected Tropical Diseases with Programs for HIV/AIDS, Tuberculosis, and Malaria. PLoS Med 3: e102.

12. Tisch DJ, Michael E, Kazura JW (2005) Mass chemotherapy options to control lymphatic filariasis: a systematic review. The Lancet Infectious Diseases 5: 514-523.

13. Reddy M, Gill SS, Kalkar SR, Wu W, Anderson PJ, et al. (2007) Oral Drug Therapy for Multiple Neglected Tropical Diseases. JAMA: The Journal of the American Medical Association 298: 1911-1924.

14. Taylor MJ, Makunde WH, McGarry HF, Turner JD, Mand S, et al. (2005) Macrofilaricidal activity after doxycycline treatment of Wuchereria bancrofti: a double-blind, randomised placebo-controlled trial. The Lancet 365: 2116-2121.

15. Stephenson LS, Kinoti SN, Latham MC, Kurz KM, Kyobe J (1989) Single dose metrifonate or praziquantel treatment in Kenyan children. I. Effects on Schistosoma haematobium, hookworm, hemoglobin levels, splenomegaly, and hepatomegaly. The American journal of tropical medicine and hygiene. pp. 436-444.

16. Stephenson LS, Latham MC, Kurz KM, Kinoti SN, Brigham H (1989) Treatment with a single dose of albendazole improves growth of Kenyan schoolchildren with hookworm, Trichuris trichiura, and Ascaris lumbricoides infections. The American journal of tropical medicine and hygiene. pp. 78-87.

17. Stephenson LS, Latham MC, Adams EJ, Kinoti SN, Pertet A (1993) Weight gain of Kenyan school children infected with hookworm, Trichuris trichiura and Ascaris lumbricoides is improved following once- or twice-yearly treatment with albendazole. The Journal of nutrition. pp. 656-665.

18. Stephenson LS, Latham MC, Adams EJ, Kinoti SN, Pertet A (1993) Physical fitness, growth and appetite of Kenyan school boys with hookworm, Trichuris trichiura and Ascaris lumbricoides infections are improved four months after a single dose of albendazole. The Journal of nutrition. pp. 1036-1046.

19. Nokes C, Grantham-McGregor SM, Sawyer AW, Cooper ES, Bundy DA (1992) Parasitic helminth infection and cognitive function in school children. Proceedings Biological sciences / The Royal Society. pp. 77-81.

20. Nokes C, Grantham-McGregor SM, Sawyer AW, Cooper ES, Robinson BA, et al. (1992) Moderate to heavy infections of Trichuris trichiura affect cognitive function in Jamaican school children. Parasitology 104: 539-547.

21. Nokes C, McGarvey ST, Shiue L, Wu G, Wu H, et al. (1999) Evidence for an improvement in cognitive function following treatment of Schistosoma japonicum infection in Chinese primary schoolchildren. The American journal of tropical medicine and hygiene. pp. 556-565.

22. Beasley NM, Tomkins AM, Hall A, Kihamia CM, Lorri W, et al. (1999) The impact of population level deworming on the haemoglobin levels of schoolchildren in Tanga, Tanzania. Tropical medicine & international health : TM & IH. pp. 744-750.

23. Bhargava A, Jukes M, Lambo J, Kihamia CM, Lorri W, et al. (2003) Anthelmintic treatment improves the hemoglobin and serum ferritin concentrations of Tanzanian schoolchildren. Food and Nutrition Bulletin. pp. 332-342.

24. Stoltzfus RJ, Chway HM, Montresor A, Tielsch JM, Jape JK, et al. (2004) Low dose daily iron supplementation improves iron status and appetite but not anemia, whereas quarterly anthelminthic treatment improves growth, appetite and anemia in Zanzibari preschool children. The Journal of nutrition. pp. 348-356.

25. Elliott A, Namujju P, Mawa P, Quigley M, Nampijja M, et al. (2005) A randomised controlled trial of the effects of albendazole in pregnancy on maternal responses to mycobacterial antigens and infant responses to bacille Calmette-Guerin (BCG) immunisation [ISRCTN32849447]. BMC Infectious Diseases 5: 115.

26. Gyapong JO, Kumaraswami V, Biswas G, Ottesen EA (2005) Treatment strategies underpinning the global programme to eliminate lymphatic filariasis. Expert Opinion on Pharmacotherapy 6: 179-200.

27. Bockarie MJ, Tisch DJ, Kastens W, Alexander ND, Dimber Z, et al. (2002) Mass treatment to eliminate filariasis in Papua New Guinea. The New England journal of medicine. pp. 1841-1848.

28. Albonico M, Bickle Q, Ramsan M, Montresor A, Savioli L, et al. (2003) Efficacy of mebendazole and levamisole alone or in combination against intestinal nematode infections after repeated targeted mebendazole treatment in Zanzibar. Bulletin Of The World Health Organization. pp. 343-352.

29. Larocque R, Casapia M, Gotuzzo E, MacLean JD, Soto JC, et al. (2006) A double-blind randomized controlled trial of antenatal mebendazole to reduce low birthweight in a hookworm-endemic area of Peru. Tropical medicine & international health : TM & IH. pp. 1485-1495.

30. Flohr C, Tuyen LN, Lewis S, Minh TT, Campbell J, et al. (2007) Low efficacy of mebendazole against hookworm in Vietnam: two randomized controlled trials. The American journal of tropical medicine and hygiene. pp. 732-736.
